# β-Elemene Synergizes With Gefitinib to Inhibit Stem-Like Phenotypes and Progression of Lung Cancer via Down-Regulating EZH2

**DOI:** 10.3389/fphar.2018.01413

**Published:** 2018-11-30

**Authors:** Haibo Cheng, Xiaoyin Ge, Shiqin Zhuo, Yanan Gao, Bo Zhu, Junfeng Zhang, Wenbin Shang, Dakang Xu, Weihong Ge, Liyun Shi

**Affiliations:** ^1^Collaborative Innovation Center of Cancer Prevention and Treatment, The First School of Clinical Medicine, Nanjing University of Chinese Medicine, Nanjing, China; ^2^School of Medicine and Life Science, Nanjing University of Chinese Medicine, Nanjing, China; ^3^School of Pharmaceutics, Zhejiang Chinese Medical University, Hangzhou, China; ^4^Faculty of Medical Laboratory Science, Ruijin Hospital, School of Medicine, Shanghai Jiao Tong University, Shanghai, China; ^5^Key Lab of Inflammation and Immunoregulation, Hangzhou Normal University School of Medicine, Hangzhou, China; ^6^Hudson Institute of Medical Research, Department of Molecular and Translational Science, Monash University, Clayton, VIC, Australia

**Keywords:** elemene, gefitinib, lung cancer, stemness, EZH2

## Abstract

The inhibitors for EGF receptor tyrosine kinase (EGFR-TKIs) such as gefitinib have been used as a standard treatment for non-small cell lung cancer (NSCLC), but the increasingly occurrence of drug resistance, the associated adverse effects and the enrichment of cancer stem cells significantly impedes its clinical application. β-elemene is a natural sesquiterpene with potent anti-cancer ability, and also it is renowned for its plant-origin, safety and the additive effect with traditional therapies, which prompt us to explore its potential to co-operate with TKIs to achieve greater therapeutic efficacy. Impressively, our study demonstrates that, elemene, in combination of gefitinib, displayed a significantly higher activity in inhibiting lung cancer cellular proliferation, migration and invasion. More importantly, combinative treatment profoundly impaired the epithelial to mesenchymal transition (EMT), the stem-like properties and the self-renewal capacity of lung cancer cells, and hence impeded the *in vivo* tumor development. We also reveal that the synergistic anti-tumor effect of elemene and gefitinib was largely mediated their regulation of enhancer of zeste homolog 2 (EZH2), an oncogenic histone methyltransferase and gene transcriptional regulator. Thus, our data indicate that combinative treatment of elemene and gefitinib has greater anti-neoplastic activity and greater efficacies in targeting cancer stem-like properties, mainly through regulating the malignant gene modifier and hence the subsequent effector molecules required for cancer progression. The findings may have potential implications for treating aggressive and resistant lung cancers.

## Introduction

Lung cancer is one of the most deadly and the most prevalent cancer worldwide, among which non-small cell lung cancer (NSCLC) accounts for approximately 85% (Chen Z. et al., 2014). In additional to the traditional treatments, such as surgery, radiotherapy and chemotherapy, the target therapy like the inhibitors for epidermal growth factor receptor-tyrosine kinase (EGFR-TKIs) has been approved as a standard first-line therapy in NSCLC patients with sensitive EGFR mutations (Maemondo et al., 2010). However, almost all patients responding to EGFR-TKIs would eventually develop drug resistance in 1 year despite the initial success, significantly impeding the therapeutic efficacy (Li et al., 2014). Moreover, accumulating evidences have indicated that chemotherapy or radiotherapy, if unable to kill cancer cells, would invariably cause the enrichment of cancer stem cells (CSCs) (Reya et al., 2001). CSCs is a small sub-population of cancer cells presumably derived from bulk tumors but adopting the stem-like traits, including the increased motility and invasiveness, the transition from epithelial to mesenchymal phenotypes, the resistance to anti-tumor drugs, and the tumor-initiating potential (Mani et al., 2008; Singh et al., 2010). The induction of stem-like cells is therefore considered as a risk factor for poor prognostics and cancer recurrence, and targeting CSCs become a novel promising therapeutic strategy (Li et al., 2017). In this regarding, combinative therapy that generally targets multiple key signaling molecules may have greater effect than the monotherapy in conquering cancer stem-like phenotypes (Morgillo et al., 2013; Wei et al., 2017).

Another major challenge facing the current cancer treatment is the high toxicity of chemotherapeutic drugs (van Meerbeeck et al., 2011). To achieve the optimal therapeutic efficacy, high doses and sustained application of chemo-drugs are required. This would invariably generate a series of side effects such as anemia, nausea, fatigue and hair loss, etc. Thus, it is of clinical relevance to find the agents that can be combined with conical TKIs or other chemotherapeutic drugs, allowing the drug use at lower dose without affecting their efficacy.

β-elemene (refer to elemene thereafter) is an active compound isolated from traditional Chinese herb Rhizoma Zedoariaem (Jiang et al., 2017). It has been approved to treat a wide spectrum of cancers including brain, breast, prostate, ovarian and lung cancers (Yao et al., 2008). Elemene has been demonstrated to exert a wide range of anti-tumor activities. It can inhibit cellular proliferation, induce progressive apoptosis, and suppress cellular invasion and metastasis (Zhang et al., 2012; Zhao et al., 2015). As a natural plant-derived agent, elemene is renowned for its safety, efficacy and less adverse effects, making it a rational candidate for co-treatment of cancer, In support of this concept, elemene has been shown to potentially reverse the chemo-resistance of cisplatin and oxaliplatin and increase drug accessibility and cellule cytotoxicity (Zhang et al., 2014; Li et al., 2016). Also, elemene was reported to enhance the effect of EGFR inhibitor in glioblastoma multiforme and reduce the resistance of gefitinib, although the exact mechanism remains largely elusive (Mu et al., 2016). Recently, the clinical trial of combination of elemene and EGFR-TKI for advanced EGFR-TKI-resistant NSCLC has been approved in China (ChiCTR-IPR-17012912 and NCT03123484), promoting further evaluation and mechanistic understanding of elemene to be as an adjuvant for the combinative therapy.

In this study, we show that the combination of elemene and gefitinib, both at lower dose, exerted the synergistic effect in inhibiting cellular proliferation, migration and invasion, enhancing the TKI sensitivity in lung cancer cells. Importantly, the combined treatment repressed cancer stem-like phenotypes, reversed the mesenchymal phenotypes and inhibited their sphere-forming ability, and consequentially, inhibited tumor development in xenograft mice. The therapeutic effect is believed to be associated with the down-regulation of the epigenetic modifier, enhancer of zeste homolog 2(EZH2) (Kim and Roberts, 2016; Wang et al., 2017). Thus, we propose that co-administration of elemene and gefitinib has remarkable antitumor potential and warrants further development as a promising therapeutic approach in treating drug-resistant NSCLC.

## Materials and Methods

### Reagents and Primers

β-elemene (99.2% purity) was purchased from the Chinese National Institutes for Food and Drug Control, with a molecular formula of C15H24 and molecular weight of 204.35 (Supplementary Figure S1A). The reagent was dissolved in dimethyl sulfoxide (DMSO; Sigma-Aldrich, St. Louis, MO, United States) at 20 mg/ml as a stock solution. Gefitinib was provided by AstraZeneca (Gaithersburg, MD, United States). The antibodies for E-Cadherin (cat. 1702-1) and N-Cadherin (cat. 2447-1) were obtained from Abcam (Cambridge, MA, United States). The fluorescein-conjugated antibodies to CD24 (cat. 555428), CD44 (cat. 555478) and the isotype controls were from BD PharMingen (San Diego, CA, United States). EZH2 and PD-L1 antibodies were purchased from Cell Signaling. The EZH2 inhibitor GSK343 was obtained from Sigma-Aldrich. The plasmid pcDNA3.1-EZH2 was provided by the Public Protein/Plasmid Library (PPPL).This Primers used in the study were included in Supplementary Tabel 1.

### Statistical Analysis

Data were expressed as mean ± standard deviation (SD) and analyzed by Student’s *t*-test or by ANOVA with the Dunnet method. Differences were considered significant at *p* < 0.05.

Detailed methods are included in Supplementary Material.

## Results

### Elemene and Gefitinib Synergistically Inhibit the Viability and Proliferation of Lung Cancer Cells

**FIGURE 1:**
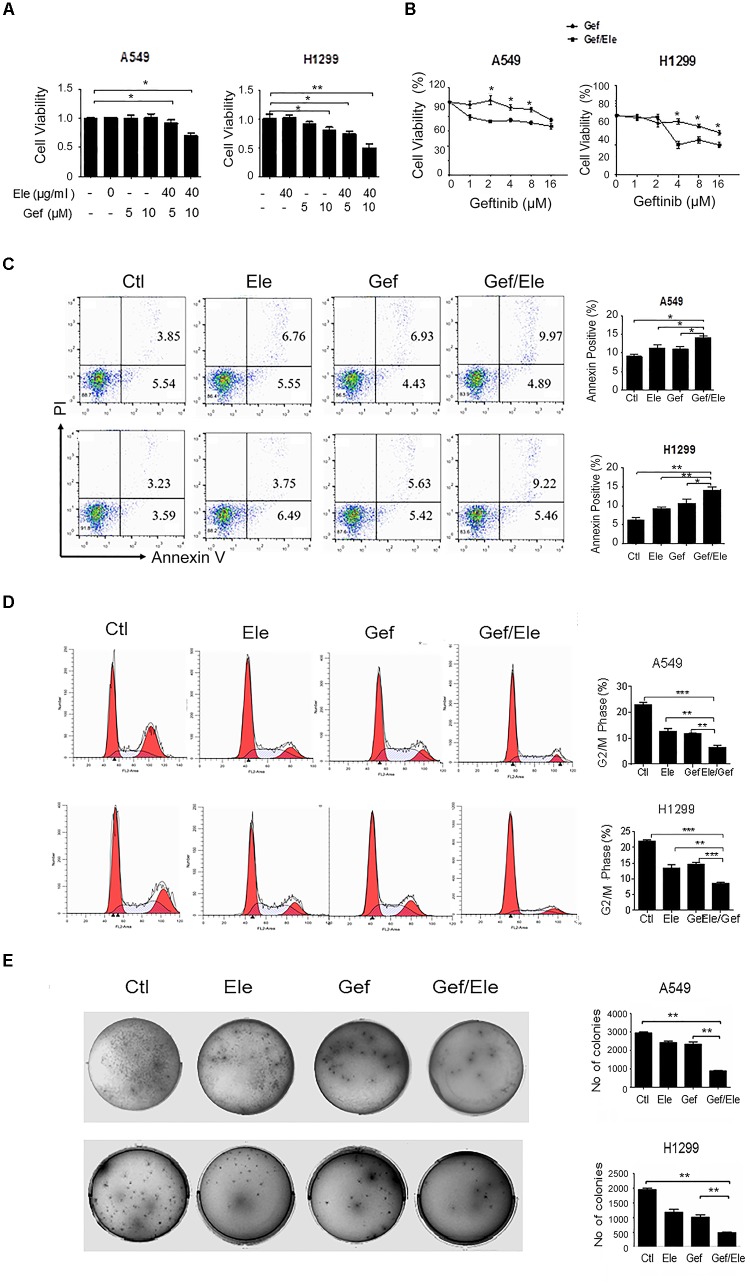
Elemene and gefitinib synergistically inhibit the viability and proliferation of lung cancer cells. **(A,B)** A549 and H1299 cells were treated with elemene, gefitinib or their combination at the indicated concentration; or cells were treated with various doses of gefitinib alone, or gefitinib in combination with elemene (40 μg/ml). Cellular viability was assessed by the MTS method 24 h post treatment. **(C,D)** A549 and H1299 cells were treated with elemene (40 μg/ml), gefitinib (5 μM) or their combination, respectively, for 24 h. Cellular apoptosis were detected with Annexin V/PI double staining followed by flow cytometry analysis, and cell cycles were detected upon PI staining. The representative images and the quantification data were shown. **(E)** A549 and H1299 cells were incubated with elemene (40 μg/ml), gefitinib (5 μM) or their combination for 14 days. Cellular colonigenic ability was examined by the colony-forming assay. Representative dishes and quantification of the colony numbers were shown. Data are representative of three independent experiments and presented as mean ± SD. ^∗^*p* < 0.05, ^∗∗^*p* < 0.01, ^∗∗∗^*p* < 0.001 by student *t’*s test.

Since high dose of anti-tumor agents is generally associated with high toxicity and severe advert effects, we sought to identify some combinative regimens that can take advantage of the synergistic effects of the agents at lower dose without affecting their efficacy. To this end, we initially applied a sublethal concentration of elemene and gefitinib to the two human cancer cell lines (Supplementary Figure S1B). It was revealed that the administration of elemene or gefitinib, at concentrations of 40 μg/ml or 5 μM, respectively, only exerted mild effects on cellular viability in both A549 and H1299 cells, the two cancer cell lines proved to be EGFR-TKI resistant (Shi et al., 2017). However, the combination of two agents caused a significant reduction in cellular viability in human lung cancer cells (Figure 1A). The addition of elemene appeared to profoundly increase gefitinib susceptibility and reduce the numbers of living cells following treatment (Figure 1B). Consistent with this, FACS analysis indicated that gefitinib, in combination with elemene, caused an elevation in cellular apoptosis in both A549 and H1299 cells (Figure 1C).

We then assessed the effect of combinative treatment on cellular proliferation, and the cell cycles of cancer cells were analyzed firstly. The result showed that, compared with either single agent, combination of elemene with gefitinib caused a marked cycle arrest in A549 and H1299 cells, inhibiting cellular entry to G2/M phase (Figure 1D). More strikingly, the colony-forming ability of lung cancer cells were significantly reduced by co-treatment of elemene or gefitinib, compared with that upon either of single agent treatment (Figure 1E). Together, our data indicated that co-treatment of elemene and gefitinib had a synergistic effect in reducing cellular viability and proliferation in lung cancer.

### Combination of Elemene and Gefitinib Exerts More Profound Effect on the Invasion and Migration of NSCLC Cells

**FIGURE 2:**
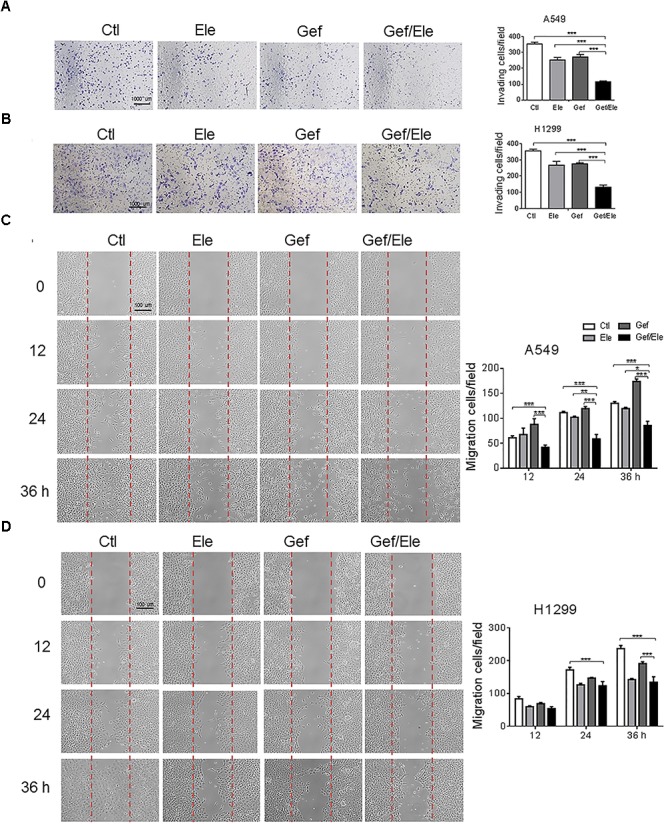
Gefitinib combined with elemene suppresses cellular invasion and migration. A549 and H1299 cells were incubated with elemene (40 μg/ml), gefitinib (5 μM) or their combination, respectively. **(A,B)** The invasive capacity of cells was detected by the Boyden chamber transwell assay. The mean numbers of cells in five fields per membrane were counted. **(C,D)** The cellular migratory capacity was analyzed by the wound healing assay. Shown are the representative photographs of scratched areas and cell migration indexes at 0, 12, 24, and 36 h post scratching. All the experiments were performed in triplicates. Data are presented as mean ± SD. ^∗^*p* < 0.05; ^∗∗^*p* < 0.01, ^∗∗∗^*p* < 0.001 by student *t’*s test.

Since cellular motility and invasion were closely related with cancer aggressiveness, we then explored the effect of the combination of elemene and gefitinib on cellular migration and invasiveness. By using the transwell invasion assays, we demonstrated that the invasive capacities of A549 and H1299 cells were significantly decreased upon co-treatment of elemene and gefitinib, compared with that upon either single agent treatment (Figures 2A,B). Likewise, elemene, in combination with gefitinib, displayed more profound effect in delaying wound closure in lung cancer cells, suggesting an additive effect of this co-treatment on cellular motility and migration (Figures 2C,D).

### Elemene and Gefitinib Synergistically Suppresses the Epithelial to Mesenchymal Transition in NSCLC Cells

**FIGURE 3:**
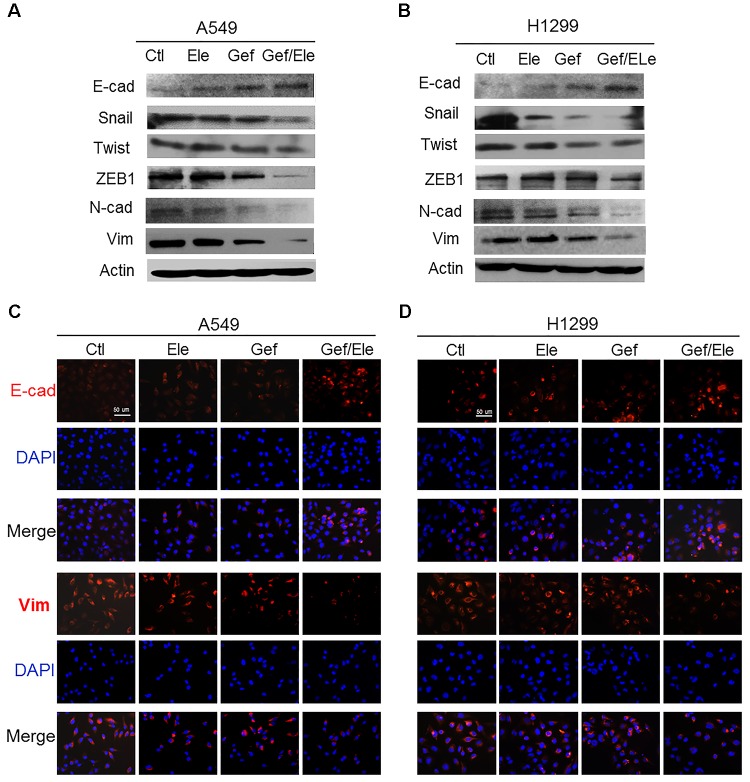
Gefitinib synergizes with elemene to reverse the EMT process in lung cancer cells. A549 and H1299 cells were treated, respectively, with elemene (40 μg/ml), gefitinib (5 μM) or their combination for 24 h. **(A,B)** Immunoblotting analysis of the representative epithelial and mesenchymal markers. **(C,D)** Immunofluorescence staining of E-cadherin (E-cad) and Vimentin (Vim). Nuclear DNA was stained with DAPI. Scale bars: 50 μm. Data are representative of three independent experiments.

Epithelial to mesenchymal transition (EMT) is a process during which cells undergo morphologic changes from epithelial to mesenchymal phenotype, allowing cells to invade and migrate and therefore contributing to cancer metastasis and progression (Garofalo et al., 2011; De Craene and Berx, 2013). As combination of elemene and gefitinib profoundly affected cellular invasion and migration in lung cancers, we wondered if the co-treatment involved in the modulation of EMT process. Clearly, our data showed that gefitinib, in combination with elemene, significantly increased the level of epithelial marker E-cadherin while suppressing the expression of mesenchymal markers (Wellner et al., 2009; Lamouille et al., 2014), in particular, ZEB1, Snail and Twist (Figures 3A,B). Immunofluorescence staining also showed that the expression of E-cadherin was enhanced while vimentin was repressed in both A549 and H1299 cells following co-treatment (Figures 3C,D). Thus, our data indicated that, compared with single agent treatment, combined therapy effectively reversed the transition from epithelial to mesenchymal phenotypes in lung cancer cells.

### Elemene and Gefitinib Synergistically Suppress the Stem-Like Properties of NSCLC Cells

**FIGURE 4:**
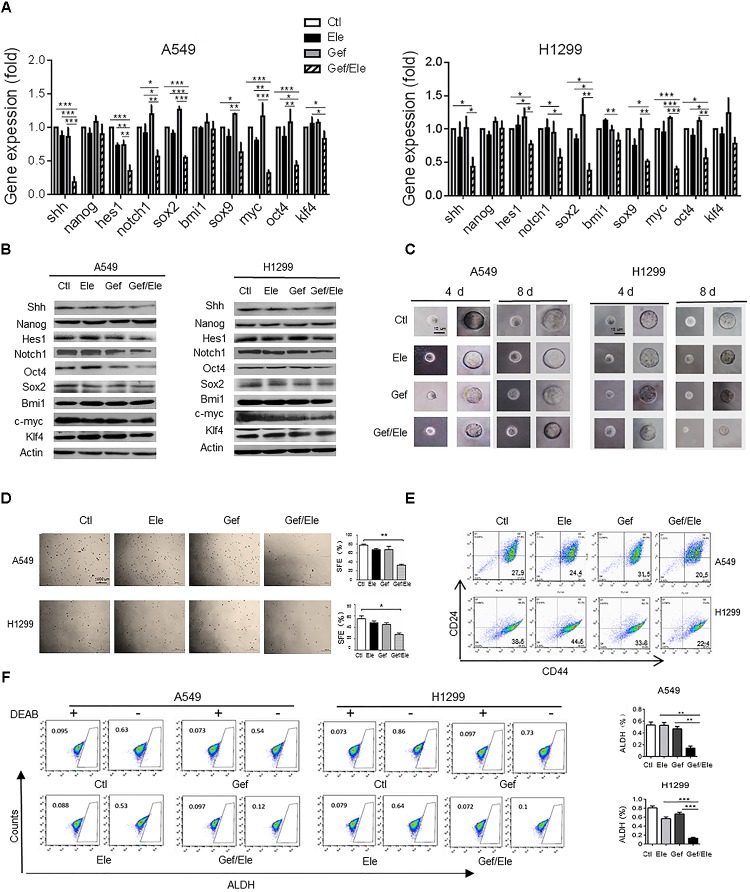
Gefitinib and elemene cooperate to repress stem-like properties of lung cancer cells. A549 and H1299 cells were treated with elemene (40 μg/ml), gefitinib (5 μM) or their combination for 24 h. **(A,B)** The relative mRNA and the protein levels of stem-associated genes were examined. **(C,D)** The cellular self-renewal capacity was assessed by the spheroid formation assay. Representative phase contrast images of self-renewal tumorspheres **(C)** and representative dishes of tumorspheres and quantification of primary spheres **(D)** were shown. Sphere-forming efficiency (SFE) was calculated as described in the section “Materials and Methods.” **(E,F)** The CD44^+^CD24^-^ or the ALDH^High^ population in A549 or H1299 cells was assessed by flow cytometry. Diethylaminobenzaldehyde (DEAB) was used as a negative control. Images are representative of three independent experiments. Data are presented as mean ± SD. ^∗^*p* < 0.05; ^∗∗^*p* < 0.01, ^∗∗∗^*p* < 0.001 by student *t’*s test.

Growing body of evidences have supported the critical link between EMT and cancer stemness. During cancer progression, the activation of EMT program renders cancer cells to acquire stem-like traits such as the self-renewal ability, the expression of stemness markers, the chemo-resistance and the *in vivo* tumorigenic potential (Chen W.J. et al., 2014). Remarkably, our data indicated that compared with either single agent, co-treatment of elemene and gefitinib caused a marked decrease in the expression of stem-related genes including sonic hedgehog (SHH), hairy and enhancer of split (HES)1, NOTCH1, c-Myc, SRY-box (SOX)2 (though to a less extent) and octamer-binding transcription factor (OCT)4 in lung cancer cells (Figures 4A,B). As a result, elemene and gefitinib synergized to impair the self-renewal ability of lung cells, as evidenced by the reduced formation of mammospheres, both in size and qualities, in A549 and H1299 cells following treatment (Figures 4C,D). Since tumor spheres were primarily composed of CSCs, this finding suggested a profound effect of the combinative treatment on CSCs. Next, we assessed the ratio of CD44^+^CD24^-/low^ cells, the subpopulation that have been demonstrated to adopt stem-like phenotypes and tumor-initiating potential (Shien et al., 2013; Chao et al., 2014). The result showed that gefitinib synergizing with elemene generated a lower percent of CD44^+^CD24^-/low^ populations among A549 or H1299 cells than either single agent (Figure 4E). Interestingly, we noted that gefitinib treatment caused an increase rather than a decrease in CD44^+^CD24^-/low^ population, implying that traditional TKI might induce the enrichment of the drug-resistant stem/progenitor cell populations. Additionally, we examined the activity of aldehyde dehydrogenase (ALDH), a class of detoxifying enzymes required for cancer chemo-resistance and aggressiveness (Ginestier et al., 2007), in cancer cells following treatment. It was revealed that, elemene, in combination with gefitinib, synergistically reduced the ALDH^high^ cell population in A549 and H1299 cells while the single agent only had mild effect (Figure 4F). Taken together, our data indicated that co-administration of elemene and gefitinib had a suppressive effect on cancer stem-like properties and reversed the malignant progression in lung cancer.

### The Synergistic Effects of Elemene and Gefitinib on NSCLC Cells Are Partially Dependent on the Down-Regulation of EZH2

**FIGURE 5:**
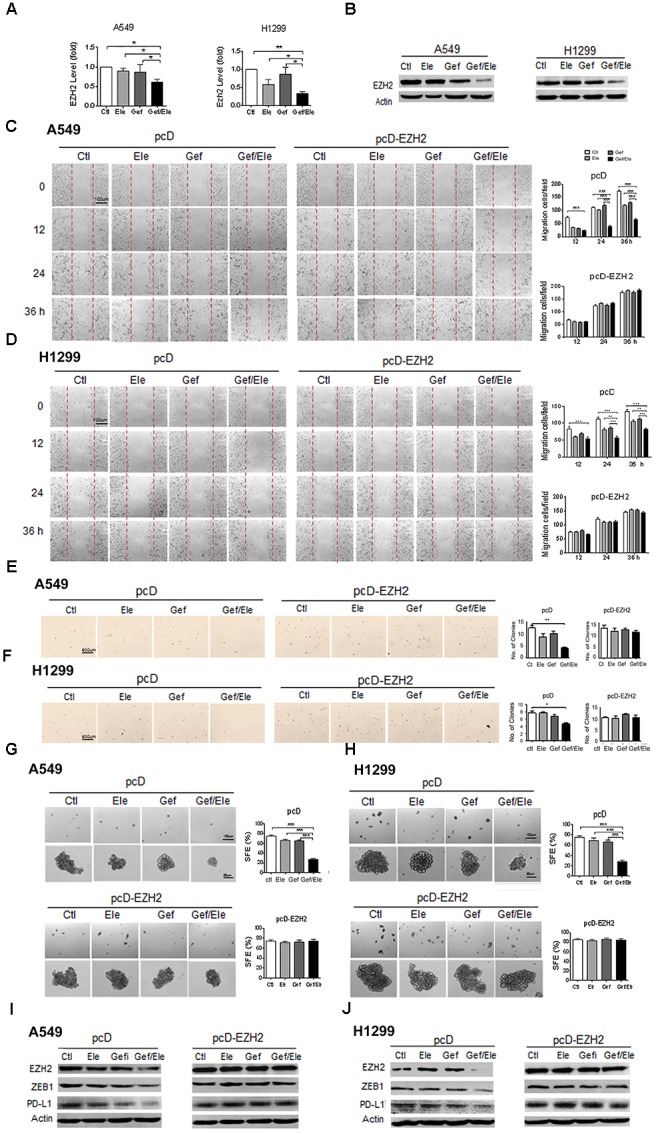
The synergistic anti-tumor effect of elemene and gefitinib is largely dependent on the regulation of EZH2. **(A,B)** The expression of EZH2 was detected by qPCR or the immunoblotting in A549 or H1299 cells treated with elemene (40 μg/ml), gefitinib (5 μM) or the combination treatment. **(C–J)** A549 and H1299 cells were transfected with EZH2-expressing or the control plasmids for 48 h, followed by the treatment of elemene (40 μg/ml), gefitinib (5 μM) or their combination. Cellular migratory capacity was examined by the scratching assay **(C,D)**. The colony formation efficiency was tested at soft agar **(E,F)**; and cellular self-renewal ability was analyzed by the sphere-forming assay **(G,H)**. The levels of EZH2, PD-L1 and ZEB1 were examined by immune-blotting **(I,J)**. Data from three independent experiments are presented as mean ± SD. ^∗^*p* < 0.05; ^∗∗^*p* < 0.01 by student *t’*s test.

Our above data showed that combination of elemene and gefitinib substantially influenced a spectrum of malignant behaviors in lung cancer cells, including cellular growth, survival, motility, invasion, and particularly, cellular EMT process and stemness-like properties. Given the multiple roles of the co-treatment involved, we speculated that the combinative therapy would operate at the upstream of the regulatory pathway that governed a panel of genes required for cancer development and progression. EZH2 is a H3K27 methyltransferase that has been recognized as a gene transcriptional regulator and a cancer driver, mainly through its ability to epigenetically and globally modify tumor-related genes (Kim and Roberts, 2016; Wang et al., 2017). Notably, our data demonstrated that compared with either of the single agent, combination of elemene and gefitinib caused a marked decrease in EZH2 levels both in A549 and H1299 cells (Figures 5A,B). Furthermore, the administration of lung cancer cells with GSK343, a well-established EZH2 inhibitor, led to a significant reduction in cellular motility and migration, indicating a functional relevance of EZH2 in our system (Supplementary Figure S2). To further understand its action mode in tumor treatment, we next introduced the EZH2-expressing plasmids into lung cancer cells following treatment. The result showed that enforced expression of EZH2 substantially reversed the inhibitory effects of combined therapy on the key malignant features such as cellular migratory ability and the colony-forming potential of cancer cells (Figures 5C–F). Moreover, the resumption of EZH2 led to an elevation in tumorsphere formation efficiency both in A549 and H1299 cells, indicating that EZH2 was critically involved in the stem-targeting effect of combinative therapy (Figures 5G,H). Additionally, we noted that the gefitinib/elemene-induced repression of ZEB1, a key mesenchymal marker gene (Chen L. et al., 2014), was increased upon EZH2 overexpression. Accompanied with this, the level of PD-L1, a prototypic checkpoint molecule essential for cancer immune evasion (Akbay et al., 2013), was also enhanced (Figures 5I,J). This implied that combinative therapy might also impact host anti-tumor immune response in a EZH2/PD-L1-dependent manner. Collectively, we herein provided the compelling evidences to show that combination of elemene and gefitinib had a greater anti-tumor activity, which was largely through its regulation of EZH2.

### Combined Treatment of Elemene and Gefitinib Impedes Cancer Development in Xenograft Mice

**FIGURE 6:**
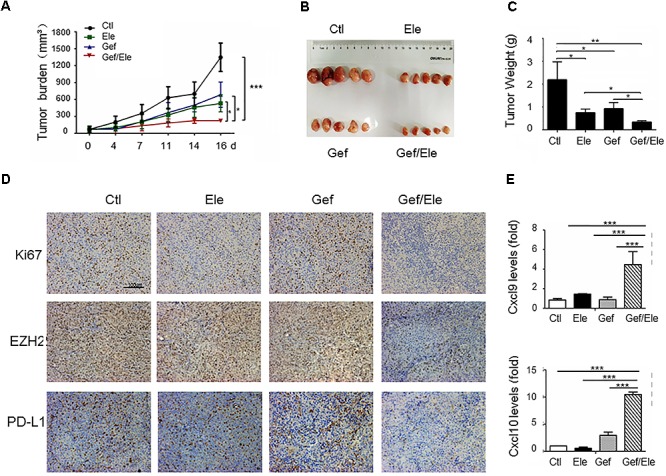
Gefitinib combines with elemene to retard tumor growth in xenograft mice. Nude mice (*n* = 5 each group) were subcutaneously (s.c.) with A549 cells (5 × 10^6^/mice) for 10 days, and then divided into four groups to receive the treatment of vehicle, elemene (40 mg/kg), gefitinib (5 mg/kg) or their combination, respectively. **(A)** Tumor volume (mm^3^) was measured twice weekly. **(B)** Macroscopic appearance of the tumors and **(C)** tumor weight at 16 d post tumor transplantation. **(D)** IHC staining of Ki67, EZH2 and PD-L1 on A549 tumor xenografts. **(E)** qPCR assay of the relative levels of CXCL9 and CXCL10 at tumors. Data are presented as mean ± SD. ^∗^*p* < 0.05; ^∗∗^*p* < 0.01, ^∗∗∗^*p* < 0.001 by student *t’*s test.

To evaluate the *in vivo* importance of combinatorial therapy, the A549 subcutaneous xenografts were generated in nude mice. Tumor-bearing animals were randomly divided into 4 groups when tumors became palpable, followed by the treatment of elemene, gefitinib or the combination, respectively. Consequentially, combination therapy resulted in a profound tumor regression in xenograft mice (82% decrease, as detected by tumor volume) while the individual treatment caused much weaker effects (about 48% decrease by gefitinib and 59% decrease by elemene) (Figures 6A,B). The analyses on tumor weights confirmed that tumor weight was more significantly reduced by drug combination compared with that upon single agent treatment (Figure 6C). Associated with this, tumor proliferation, as assessed by Ki-67 staining, was more profoundly inhibited by combined therapy compared with single agent treatment. Notably, the level of EZH2, concurrent with PD-L1, was repressed by co-treatment of elemene and gefitinib (Figure 6D). The finding was consistent with the *in vitro* observation we described above (Figures 5I,J). Interestingly, we noted that the levels of CXCL9 and CXCL10, the two conical chemokines required for efficient T cells infiltration, were remarkably up-regulated in mice receiving combined treatment that receiving single agent treatment (Figure 6E). The finding was congruent with the recent report that Th1 chemokines were susceptible to the EZH2-mediated epigenetic regulation (Peng et al., 2015) and the co-treatment likely caused the de-repression of chemokines from EZH2-dependent restraint by suppressing its expression. Consistently, we observed that the numbers of CD4^+^ and CD8^+^ T cells were increased in the gefitinib/elemene-treated mice (Supplementary Figure S3). Taken together, gefitinib, in combination with elemene, potentially repressed lung cancer development in xenograft mice, exhibiting its therapeutic value in devastating NSCLCs.

## Discussion

Lung cancer is one of the most prevalent and most deadly cancers worldwide. EGFR-TKI have been used as a first-line treatment against lung cancer, but the increasing occurrence of drug resistance and the associated adverse sides precludes it widespread application (Maemondo et al., 2010; van Meerbeeck et al., 2011; Chen Z. et al., 2014). Elemene is a novel anticancer drug known for its broad anti-tumor effects and lower adverse effects both *in vitro* and *in vivo* (Yao et al., 2008; Zhang et al., 2012, 2014; Zhao et al., 2015; Li et al., 2016; Jiang et al., 2017). It has been authorized to treat a panel of solid tumors including lung cancer, but its potential to be as an adjunctive agent for the current standard anti-tumor drug is yet to be explored (Zhang et al., 2012). In the present study, we for the first time demonstrated that administration of elemene significantly enhanced cellular responses to the canonical EGFR-TKI gefitinib, inhibiting cellular proliferation and inducing progressive apoptosis in lung cancer cells. Moreover, combination of elemene and gefitinib suppressed stem-like features in NSCLC cell lines, and reduced tumorigenic potential in xenograft mice. This anti-tumor capability was partially at least, through the disruption of EZH2-dependent oncogenic pathway. We have thus revealed a novel combinative strategy that displays a greater anti-oncogenic activity, and more importantly, it shows a great promise to overcome drug resistance and improve the current treatment for devastating lung cancer.

Evidences have shown that cancer development is a multi-step process that disturbs multiple signaling pathways essential for normal cell activity. The aberrant activation of the tumorigenic pathway like EGFR-driven signaling is frequently intertwined with other oncogenic pathways, such as the signaling driven by VEGF, RAS and PI3-K/Akt (Martinelli et al., 2010; Seguin et al., 2014). In this sense, combinative treatment that simultaneously targets the key signaling molecules would outperform a single agent to fight against cancer cells (Vujic et al., 2015). In our current study, the combination of elemene with gefitinib showed greater effect in restraining cellular proliferation, survival, invasion and migration, the core malignancies associated with EGFR signaling. Our data showed that the addition of elemene boosted cellular response to the conical TKI both in A549 and H1299 cells, the cell lines proved to be refractory to TKI therapy. This finding is of critical clinic implication since drug resistance has become a bottleneck issue for cancer therapy. Combined treatment of elemene and gefitinib may offer a novel strategy for the resistant lung cancer.

Previously, the “synergistic” strategies were primarily focused on the induction of cellular death and tumor eradication. However, the unselective cytotoxicity of the agents would invariably generate serious adverse effects and cause the treatment intolerance among patients, which would eventually come to therapy cession or suspension (Zhou et al., 2009; Sequist et al., 2011). Furthermore, accumulating data demonstrated that cancer treatment would give rise to a minor subpopulation of cells within tumors, called CSCs or cancer initiating cells (CICs) (Chen et al., 2012; Chen Z. et al., 2014). These residual post-treatment populations are relatively resistant to cytotoxic chemo-and radio-therapies and adopt more aggressive phenotypes, contributing to cancer metastasis and recurrent. Based on these two critical issues associated with cancer therapy, we firstly lowered the concentration of elemene and gefitinib to such an extent that either of them exerted only mild cytotoxic effect on lung cancer cells, but the combinative treatment showed greater anti-tumor activity and enhanced TKI sensitivity. Secondly, given the importance of CSCs in tumor initiation and progression, targeting cancer stemness becomes a primary goal for our combinative strategy. As shown in our data, gefitinib, in combination with elemene, significantly repressed cancer EMT process, inhibited the expression of stemness-associated genes, and reduced cellular self-renewal potential. Along with this, the enrichment of CD44^+^CD24^-^ or ALDH^High^ cell populations was reduced by the co-treatment in A549 and H1299 cells. Since the EMT program is closely associated with invasive and migratory capacity of cancer cells, and the enrichment of CD44^+^CD24^-^ or ALDH^High^ cells is thought to be a major event prerequisite for tumor initiation and chemo-resistance, the finding about the profound effect on stem-related features may open a new perspective on combinative treatment. The dual effect of the co-treatment, both enhancing TKI responsiveness and preventing TKI-induced stem-like progression, may make it an ideal strategy to optimize the treat efficacy and minimize the side-effect profiles (Hirsch et al., 2009; Lau et al., 2014; Van Roosbroeck et al., 2017).

In a sought to identify the mechanism responsible for the synergistic effect of the combined treatment, our data demonstrated a striking reduction of EZH2, the epigenetic regulator as well as a cancer driver, by the combinative treatment. EZH2 is a histone methyl transferase subunit of polycomb repressive complex 2 (PRC2) that is proposed to remold chromatin configure and hence modulate gene expression profile (Souroullas et al., 2016). Evidences have demonstrated that EZH2 is capable of reprogramming the gene program essential for the proliferation, stemness and metastasis of cancer cells (Kim and Roberts, 2016; Wang et al., 2017). It has been proved to highly express in a wide variety of human cancers and is regarded as a diagnostic and prognostic marker for poor outcome (Behrens et al., 2013). A recent study using a genetically engineered mouse model confirmed the oncogenic effect of EZH2 and showed that overexpression of EZH2 resulted in aberrant spread of H3K27me3 at the known tumor suppressors in lung cancer, thus pointing to EZH2 as a pressing target for cancer therapy (Zhang et al., 2016). Consistent with our current study, EZH2 inhibitors have been able to repress tumorigenesis and malignant progression. Notably, our data showed that enforced expression of EZH2 largely abrogated the anti-tumor effect of the combination of elemene and gefitinib, indicating that EZH2-dependent mechanism is critically involved in the action of combinative treatment. Moreover, since much effort has been invested to develop drug-like EZH2 inhibitors for cancer treatment, our finding about the EZH2-targeting co-treatment may offer an efficient and relatively safe strategy for cancer treatment (Kim and Roberts, 2016; Jin et al., 2017).

Additionally, our data also showed that combination of elemene and gefitinib caused a marked decrease in PD-L1 *in vitro* and *in vivo*, in parallel to the reduction of EZH2. Ectopic expression of EZH2 abolished the inhibitory effect of co-treatment and elevated PD-L1 level, implying that EZH2 had an essential role in promoting PD-L1 expression. This observation is congruent with the very recent report showing a correlation between EZH2 and PD-L1 level on cancer cells (Zingg et al., 2017). Also, we found that the expression of Th1-type chemokines CXCL9 and CXCL10, the molecules presumably subjected to EZH2-dependent epigenetic modification (Martinelli et al., 2010; Nagarsheth et al., 2016), was up-regulated upon combinative therapy. Since CXCL9 and CXCL10 constitute two major molecules for effector T cells infiltration, it is speculated that combination of elemene and gefitinib might have an additional effect on enhancing anti-tumor T cell response. Further studies might be needed to explore the potential role for combinative therapy in enhancing anti-cancer immunity.

Taken together, the combination of gefitinib and elemene exhibits the remarkably enhanced anti-cancer activity, with potential to overcome EGFR-TKI resistance, to inhibit cancer stemness and to repress cancer development capability in xenograft mice. We thus propose that this combinative therapy may become a novel promising strategy for lung (and other potentially) cancer treatment.

## Ethics Statement

All of the animal experiments were performed in accordance with the National Institutes of Health Guide for the Care and Use of Laboratory Animals, and with the approval of Animal Care and Use Committee of Nanjing University of Chinese Medicine.

## Author Contributions

LS designed the study. HC, XG, SZ, YG, and JZ performed the experiments and analyzed the data. YG, BZ, and LS wrote the manuscript. WS and DX contributed to the experimental material and provided the insightful suggestions. LS and WG supervised the program.

## Conflict of Interest Statement

The authors declare that the research was conducted in the absence of any commercial or financial relationships that could be construed as a potential conflict of interest. The reviewer EL and handling Editor declared their shared affiliation at the time of review.
