# Supplementary material for: β-Elemene Synergizes With Gefitinib to Inhibit Stem-Like Phenotypes and Progression of Lung Cancer via Down-Regulating EZH2
Source: Front Pharmacol. 2018 Nov 30;9:1413. doi: 10.3389/fphar.2018.01413 (PMC6284059; doi:10.3389/fphar.2018.01413)
Supplement: Supplementary file 1 [file Data_Sheet_1.PDF]

β-elemen synergizes withe Gefitinib to inhibit stem-like phenotypes and progression of lung cancer via downregulating EZH2

Haibo Cheng, Xiaoyin Ge, Shiqin Zhuo, Yanan Gao, Bo Zhu, Junfeng Zhang, Wenbin Shang, Dakang Xu, Weihong Ge, Liyun Shi

Table S1. qPCR primer sequences

Genes		Primers (5’->3’)
SHH	Forward	CTCGCTGCTGGTATGCTCG
	Reverse	ATCGCTCG GAGTTTCTGGAGA
NANOG	Forward	CCCCAGCCTTTACTCTTCCTA
	Reverse	CCAGGTTGAATTGTTCCAGGTC
HES1	Forward	CCTGTCATCCCCGTCTACAC
	Reverse	CACATGGAGTCCGCCTAA
NOTCH1	Forward	TGGACCAGATTGGGGAGTTC
	Reverse	GCACACTCGTCTGTGTTGAC
SOX2	Forward	GCCCGAGTGGAAACTTTTGTCG
	Reverse	GGCAGCGTGTACTTATCCTTCT
BMI1	Forward	CGTGTATTGTTCGTTACCTGGA
	Reverse	TTCAGTAGTGGTCTGGTCTTGT
SOX9	Forward	AGCGAACGCACATCAAGAC
	Reverse	CTGTAGGCGATCTGTTGGGG
MYC	Forward	GGCTCCTGGCAAAAGGTCA
	Reverse	CTGCGTAGTTGTGCTGATGT
OCT4	Forward	CTTGAATCCCGAATGGAAAGGG
	Reverse	GTGTATATCCCAGGGTGATCCTC
Actin	Forward	CTCATGAAGATCCTGACCGAG
	Reverse	AGTCTAGAGCAACATAGCACAG

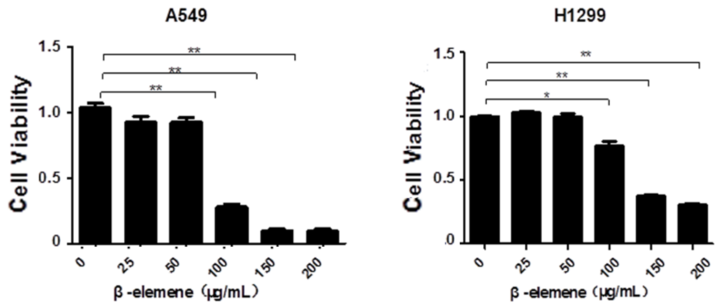

Figure S1. The effect of elemene on cellular viability of human lung cancer cells. A549 and H1299 cells were treated with elemene at the indicated concentration. Cellular viability, relative to the untreated controls, was assessed by the MTS method 24 h post treatment. The experiments were performed in triplicates. Data are presented as mean ± SD. * $P < 0.05$, ** $P < 0.01$ by student t's test.

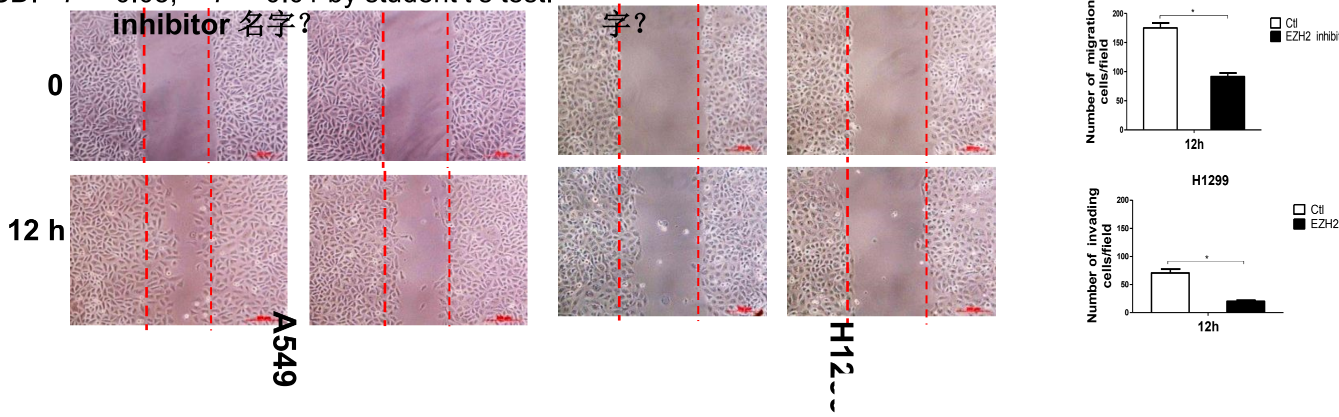

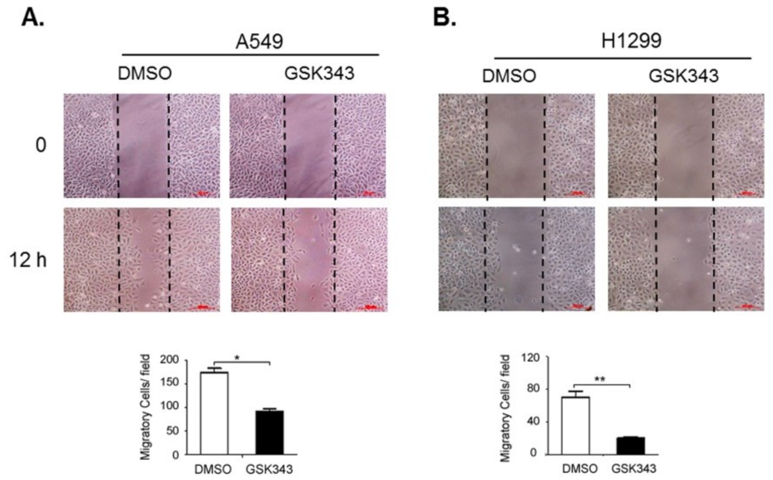

Figure S2. The effect of EZH2 inhibitor on the migratory capacity of lung cancer cells. A549 and H1299 cells were Pretreated with DMSO or GSK343 (5 μM) for 30 min, and subjected to the wound healing assay for migratory capacity. Shown were the representative photographs of scratched areas and the cell migration indexes at 0 and 12 h after wounding. The experiments were performed in triplicates. Data are presented as mean ± SD. * $P < 0.05$; ** $P < 0.01$ by student t's test.

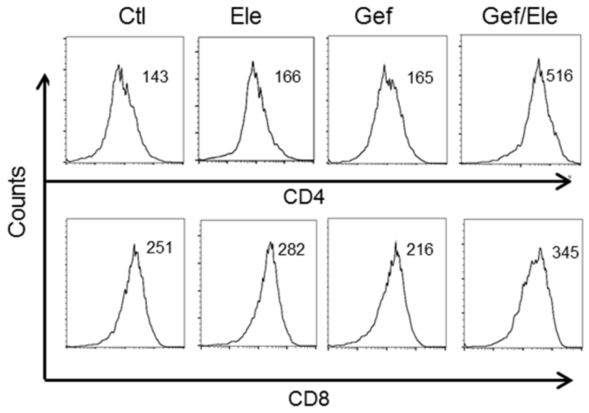

Figure S3. Combination of gefitinib and elemene enhances T cell response. BALB/c mice (n=5 each group) were subcutaneously injected with A549 cells (5×10^6/mice), followed by the treatment of the vehicle, elemene (40 mg/kg), gefitinib (5 mg/kg) or their combination. 16 d later, mice were sacrificed and tumors were collected. CD4 and CD8 positive cells were stained and analyzed by flow cytometry. Shown are the representative histogram and mean fluorescence intensity (MFI).
